# Effect of Hydrophobic Nano-SiO_2_ Particle Concentration on Wetting Properties of Superhydrophobic Surfaces

**DOI:** 10.3390/nano12193370

**Published:** 2022-09-27

**Authors:** Lei Xing, Tian Xia, Qiaoxin Zhang

**Affiliations:** School of Mechanical and Electronic Engineering, Wuhan University of Technology, Wuhan 430070, China

**Keywords:** superhydrophobic surfaces, nano-SiO_2_ particles, concentration, wettability

## Abstract

As a unique surface wettability, superhydrophobicity has great application value. A variety of preparation methods for superhydrophobic surfaces have been reported, which have the disadvantages of high cost and complicated process. In order to design a method that is easy to operate, low-cost, and suitable for large-scale preparation of superhydrophobic surfaces, in this paper, hydrophobic nano-SiO_2_ particles are used as spray fillers, and superhydrophobic surfaces are successfully obtained by the spraying process. According to the classical Cassie and Wenzel theory, the influence of the concentration change of hydrophobic nano-SiO_2_ particles on their wettability is explained, and the appropriate spray concentration parameters are obtained. The results show that the proportion of hydrophobic nano-SiO_2_ particles is lower than 0.05 g/mL, which will lead to insufficient microstructure on the surface of the coating, and cannot support the droplets to form the air bottom layer. However, an excessively high proportion of hydrophobic nano-SiO_2_ particles will reduce the connection effect of the silicone resin and affect the durability of the surface. Through theoretical analysis, there are Wenzel state, tiled Cassie state, and stacked Cassie state in the spraying process. When the substrate surface enters the Cassie state, the lower limit of the contact angle is 149°. This study has far-reaching implications for advancing the practical application of superhydrophobic surfaces.

## 1. Introduction

Superhydrophobicity is an extreme surface property whose static contact angle of the surface liquid is greater than 150° [1,2,3,4,5]. The lotus leaf is the most representative biological model in the field of superhydrophobicity. However, the mechanism of keeping the lotus leaf clean was not known until the development of scanning electron microscopy (SEM) in the mid-1960s. The secret of the lotus leaf is “coming out of the silt without being stained”. In 1977, Barthlott and Neinhuis studied the surface structure of the lotus leaf by scanning it with an electron microscope [6,7]. There is a layer of nano-scale hair-like structures on the surface of lotus leaves, and the air between the hair gaps forms an air cushion between the hair-like structures and the water. The tension of the water surface also contributes. Therefore, when water falls on the lotus leaf, it exists in the form of water droplets. When the leaf surface is tilted slightly, the water droplets roll off the leaf surface, taking away the dust. After testing, the static contact angle of water on the lotus leaf is about 164° [8,9]. The superhydrophobic surface with this “lotus leaf effect” has wide application value in the fields of anti-corrosion, anti-icing, anti-fog, oil-water separation, self-cleaning, and underwater drag reduction [10,11,12,13,14,15].

At present, the preparation methods of superhydrophobic surfaces mainly include plasma etching, laser etching, electrochemistry, and vapor deposition [16,17,18,19]. The plasma etching and the laser etching methods use high energy to generate plasma sputtering or use a laser to etch the substrate [20,21]. The electrochemical method needs to energize the substrate, and use the ionization of the polymer solution to deposit on the substrate to form a micro-nano structure [22]. Vapor deposition is currently mainly used to prepare carbon nanotubes [23]. Such methods require special equipment, which is not conducive to large-area processing, and will cause damage to the substrate. Therefore, it is of great practical significance to further study the superhydrophobic surface with a simple preparation process and wide application range.

The spray technique sprays nanoparticles and low surface energy solutions directly onto the surface to form a superhydrophobic surface. By spraying a mixed solution of nanoparticles and low-surface-energy substances, the surface has both nano-scale protrusions and low surface energy, so that a superhydrophobic surface can be easily and quickly obtained [24,25,26]. This method has a wide range of applications, can be processed in a large area, and has great advantages in the preparation of superhydrophobic surfaces. Gong et al. [27] obtained a surface with excellent superhydrophobicity on an aluminum alloy substrate by spraying with F-SiO_2_ (SiO_2_ nanoparticles treated with 1H, 1H, 2H, 2H-Perfluorodecyltriethoxysilane fluorination), epoxy resin adhesive, fluorosilicone varnishes, and white fluorinated polyurethane coatings. Results showed that superhydrophobic coating exhibited excellent water-repellency. The static contact angle of the superhydrophobic coating is 161.35°. Kim et al. [28] prepared coatings with hydrophobic nano-silica particles and siloxane binders and used them to prepare microstructured surfaces by a simple spraying method, obtaining a superhydrophobic surface with a static contact angle of about 160°. Guo et al. [29] sprayed the nano-silica dispersion on the semi-cured silicone-modified polyurethane resin by a two-step spraying method and successfully prepared a superhydrophobic coating with a static contact angle of 159°.

It can be seen that a superhydrophobic surface with a high static contact angle can be constructed by spraying technology, but the literature does not mention how the concentration of hydrophobic nano-SiO_2_ particles in spraying affects the superhydrophobicity of the surface. In this paper, a combination of theory and experiment was used to study the effect of the concentration of hydrophobic nano-SiO_2_ particles in the spray coating on the superhydrophobicity of the surface, and a better spray concentration parameter was obtained. The three states in the spraying process were analyzed theoretically, and the lowest static contact angle after the droplet enters the Cassie wetting state was calculated. This study has far-reaching implications for advancing the practical application of superhydrophobic surfaces.

## 2. Materials and Methods

### 2.1. Materials

**Figure 1:**
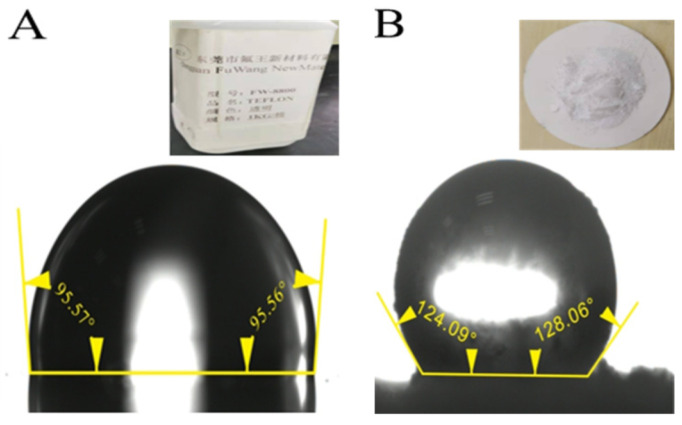
The static contact angle data of the aluminum alloy polished flat substrate sprayed with FW-8800 and hydrophobic nano-SiO_2_ particles. The illustration is the real object. (**A**) FW-8800; (**B**) hydrophobic nano-SiO_2_ particles.

Silicone resin coating FW-8800 is a low surface energy coating produced by Fluoroking New Materials Co., Ltd. (Dongguan, China). Its main component is siloxane polymer. The coating has good temperature resistance, acid resistance, and corrosion resistance, and the main structure forming the molecular structure is the siloxane bond, which has strong bond energy and high thermal decomposition temperature. In an environment of less than 150 °C, the coating does not undergo thermal decomposition, aging, and discoloration. It has excellent UV resistance and weather resistance, so it has good weather resistance, the phenomenon of yellowing and chalking of the coating film is slight, and the characteristics of the coating film can be maintained for a long time. It also has excellent adhesion to various metal substrates, wood, and stone. As shown in Figure 1A, the static contact angle obtained by spraying FW-8800 on the aluminum alloy polished flat substrate alone was about 95.6°. The hydrophobic nano-SiO_2_ particles purchased from Yuanjiang Chemical Co., Ltd. (Shanghai, China) were used, and the particle size was 50 nm. The hydrophobic nano-SiO_2_ particles were obtained by modifying the hydrophilic nano-SiO_2_ particles with silane or siloxane. Its static contact angle was 124°~128° (Figure 1B). the hydrophobicity of the hydrophobic nano-SiO_2_ particles will remain stable for a long time, which has important implications for the long-term durability of superhydrophobic surfaces.

### 2.2. Methods

#### 2.2.1. Preparation before Spraying

We used 500# and 1000# sandpapers to polish the 5052 aluminum alloy substrate in turn. The surface-treated aluminum alloy plate was ultrasonically cleaned with acetone, ethanol, and deionized water, then placed in an oven at 80 °C for drying. Since the viscosity of the coating should not be too high during the spraying process, 100 mL silicone resin (Fuwang New Material Co., Ltd., Dongguan, China) and absolute ethanol were mixed at 1:1, and then hydrophobic nano-SiO_2_ particles with a concentration (0.025–0.1 g/mL) were added (Yuanjiang Chemical Technology Co., Ltd., Shanghai, China. Particle size 50 nm). After 20 min of magnetic stirring and 20 min of ultrasonic dispersion and mixing, a uniformly dispersed suspension of hydrophobic nano-SiO_2_ particles was obtained, which was loaded into the spray gun (Fujiwara Tools Co., Ltd., Taizhou, China. Model: W-71-1G). The ambient temperature during spraying was 20~25 °C.

#### 2.2.2. Preparation of Superhydrophobic Surfaces

The above-mentioned 5052 aluminum alloy substrate was placed under the spray gun frame. The height of the spray gun frame was 25 cm from the horizontal ground, the spray pressure was 0.6 MPa, the paint flow rate was 3 g/min, the gun speed was 0.2~0.3 m/s, and the spraying angle was 90°. Under the above spraying process parameters, superhydrophobic surfaces with different concentrations of hydrophobic nano-SiO_2_ were successively obtained.

### 2.3. Characterization

The surface morphology of the prepared samples was detected by a JSM-IT300 scanning electron microscope (JEOL Co., Ltd., Tokyo, Japan). Its main technical indicators are (1) Secondary electron image resolution: 3.0 nm. (2) Backscattered electron image resolution: 4.0 nm. (3) Magnification range: 5 times to 300,000 times (continuously adjustable). (4) Accelerating voltage: 0.3~30 kV (multi-level adjustable). The KEYENCE VK-250K laser confocal microscope (KEYENCE Co., Ltd., Osaka, Japan)was used to characterize the three-dimensional topography of the superhydrophobic surface. The laser confocal microscope can obtain the microscopic topography at different depths by fast scanning imaging.

The surface wetting properties were characterized by measuring the static contact angle of water droplets. The surface static contact angle was measured by SDC-80 optical contact angle measuring instrument (Shengding Precision Instrument Co., Ltd., Dongguan, China). The reliability of the measurement data was ensured by measuring the contact angle of the five regions of the sample. The contact angle was obtained by taking pictures of water droplets and analyzing them through software ImageJ-v1.8.0 (National Institutes of Health, Bethesda, MD, USA).

## 3. Results and Discussion

### 3.1. Surface Topography

**Figure 2:**
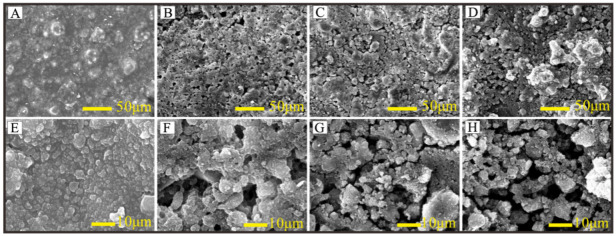
Surface topography of polished planes sprayed with different concentrations of hydrophobic nano-SiO_2_ coatings. (**A**,**E**) 0.025 g/mL; (**B**,**F**) 0.05 g/mL; (**C**,**G**) 0.075 g/mL; (**D**,**H**) 0.1 g/mL.

Figure 2 shows the surface topography of the polished plane under the spraying of the hydrophobic nano-SiO_2_ coating at different concentrations. Figure 2A–D are the surface topography of the polished plane under the spraying of the hydrophobic nano-SiO_2_ coating with a concentration of 0.025–0.1 g/mL, while Figure 2E–H are the corresponding enlarged images of the surface topography. The mechanical properties of the coating are determined by the microstructure. When the concentration is 0.025 g/mL, the surface of the sample has a continuous film layer, basically no pores and defects, the larger protrusions are large particles formed by agglomeration of SiO_2_, and there are submicron protrusions on the large particles. The microspheres are in the Wenzel wet state, and the microstructure of the surface is not enough to support the droplets to form a superhydrophobic surface. When the concentration is 0.05 g/mL, the proportion of film-forming resin decreases, and pores begin to appear on the surface of the film layer. After that, the surface in the subsequent concentration range is in a Cassie-wet state and has a certain superhydrophobicity. When the concentration is 0.075 g/mL, the proportion of film-forming resin is further reduced, and cracks are formed in the plane. When the concentration is 0.1 g/mL, there are only rifts formed by the aggregation of nano-SiO_2_ particles in the microscopic morphology, as well as sub-micron particles. As the concentration increases, the content of film-forming substances decreases, and the surface morphology tends to be large particles formed by the accumulation of nano-SiO_2_ particles. The ability of the plane to store air becomes stronger, the solid-liquid contact area is further reduced, the static contact angle increases macroscopically, and the superhydrophobicity of the surface is enhanced, but the mechanical properties of the connection are weakened due to the lack of film-forming substances.

**Figure 3:**
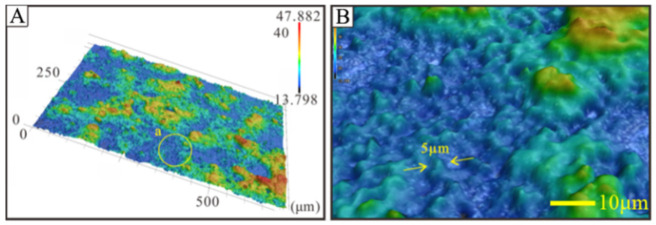
3D topography of the polished base coating surface. (**A**) After 15 sprays; (**B**) enlarged image.

Figure 3 shows the 3D surface profile of the polished coating surface after 15 sprays. It can be found that the rubbed polished surface is completely covered by the sprayed coating. The surface of the coating has a granular protruding microstructure similar to a lotus leaf, with a size of about 5 μm. These microstructures can effectively encapsulate air, thereby reducing the contact area between the liquid and the solid surface, reducing the adhesion between the solid-liquid interface, and finally achieving the effect of enhancing hydrophobicity.

### 3.2. Surface Wettability

**Figure 4:**
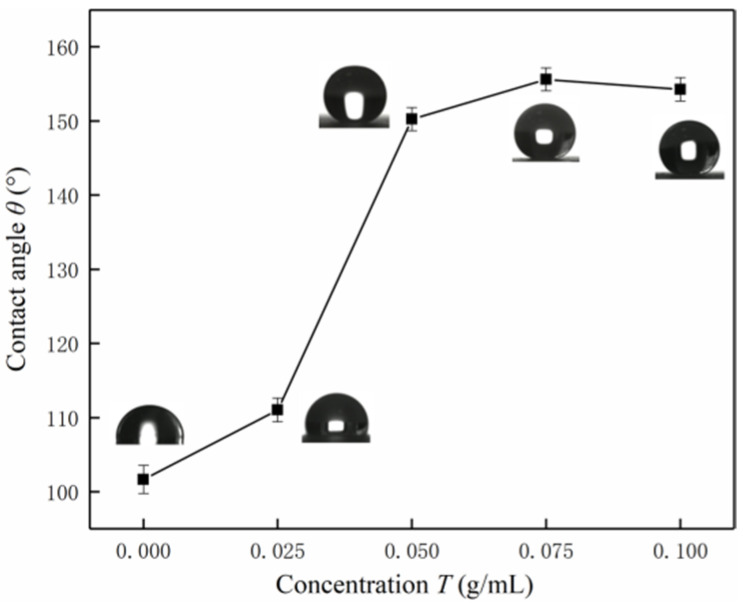
Contact angle data of different concentrations of SiO_2_ coatings sprayed.

Figure 4 is a data graph of wetting performance under spraying of SiO_2_ coatings with different concentrations. When the hydrophobic nano-SiO_2_ particle concentration of the coating gradually increases to 0.025 g/mL, the contact angle of the sample increases. This is due to the gradual increase in the number of nanoparticles acting as microstructure supports, and the corresponding increase in the gas-liquid contact area, which is in the range of the contact angle described by the theoretical Wenzel state. When the concentration of the sprayed coating on the substrate reaches 0.05 g/mL, the change of the contact angle is large; when it exceeds 0.05 g/mL, the change is small, and the concentration reaches saturation.

**Figure 5:**
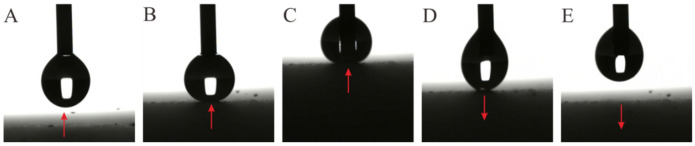
The experimental process diagram of the surface adhesion of the polished base coating. (**A**~**C**) The process of moving up the test bed; (**D**,**E**) the process of moving down the test bed.

The adhesion test process of the polished substrate sample sprayed at a concentration of 0.05 g/mL is shown in Figure 5. Figure 5A volume of 5 μL of droplets is output from the infusion needle, and the superhydrophobic sample gradually approaches the droplets as the test bench rises. During this process, it can be seen that when the test bed moves up in the direction of the red arrow, it makes contact with the droplet when it reaches the position of Figure 5B. It continues to move upward as the liquid is squeezed, and when it reaches the position shown in Figure 5C, the test bed moves downward (as shown in Figure 5D,E). When the droplet is separated from the sample, there is no residual droplet on the surface of the sample, indicating that the sample has low adhesion to the droplet.

### 3.3. Wetting Mechanism

**Figure 6:**
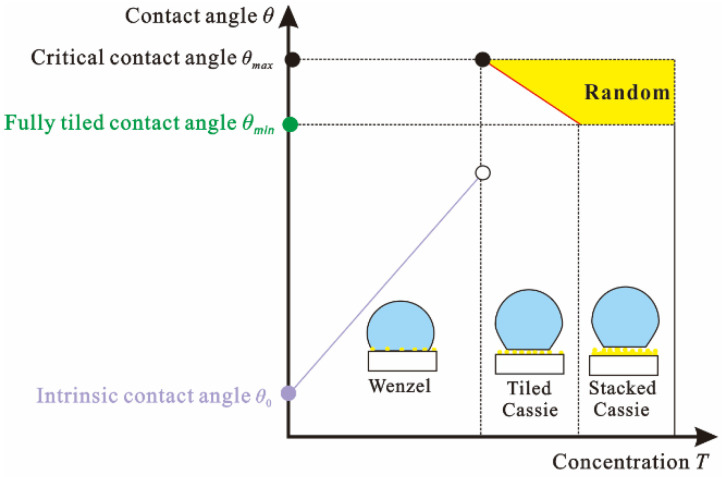
Effect of hydrophobic nano-SiO_2_ particle concentration on wetting state.

The microstructure of the hydrophobic nano-SiO_2_ particles as the supporting droplets is attached to the surface of the substrate through the connection of film-forming substances. With the increase of the concentration, the wetting performance of the sprayed surface can be divided into three states, as shown in Figure 6:

When the concentration of nano-SiO_2_ particles is low, the distance between the microspheres on the polished surface is too large, the linear density of the three-phase contact is too low, and the surface tension provided by the micro-nano structure cannot support the droplets, resulting in the infiltration of the droplets. With the increase of the nano-SiO_2_ particle concentration, the contact angle of the droplet increases with the increase of the surface roughness, which belongs to the Wenzel state. At this time, based on Wenzel’s theory [30], the relationship between the contact angle and the concentration of nano-SiO_2_ particles is as follows:

(1) $$\cos\theta_{w}=S_{1}\left(T\right)\cos\theta_{0}/S_{2}$$

where *θ_w_*, *θ*_0_, *S*_1_, and *S*_2_ represent the apparent contact angle in the Wenzel state, intrinsic contact angle, actual solid-liquid contact area of the rough surface, and apparent solid-liquid contact area, respectively. The three-phase contact line density is an important index to measure the transition of the wetting state. According to the research conclusions of Extrand [31], the calculation formula of the critical value of the three-phase contact line density is:

(2) $$\Lambda_{c}=\frac{-\rho gV^{1/3}{\left\{\tan\left(\theta_{a}/2\right)\left[3+\tan^{2}\left(\theta_{a}/2\right)\right]\right\}}^{2/3}}{\left[{\left(36\pi \right)}^{1/3}\gamma \cos\theta_{a}\right]}$$

where *ρ* is the density of the droplet, which is 1000 kg/m^3^;  g is the acceleration of gravity, which is 9.8 N/kg; *V* is the volume of the droplet, which is 5 μL; *θ_a_* is the advancing contact angle, which is 128°; and γ is the gas-liquid surface tension, which is 72 mN/m. The final *Λ_c_* is 0.471.

Equation (2) Tiled Cassie state: When the critical value of the three-phase contact line density is the most ideal microstructure distribution state, the gas-liquid contact area fraction is the largest, and the contact angle is also largest at this time. However, with the continuous increase of the concentration of hydrophobic nano-SiO_2_ particles, the three-phase contact line density on the surface continues to increase, the gas-liquid contact area fraction decreases, and the corresponding solid-liquid contact area fraction increases, resulting in a smaller droplet contact angle. Based on Cassie’s theory [32], the mathematical relationship is as follows:

(3) $$\cos\theta_{c}=f_{1}\left(T\right)\cos\theta_{0}+f_{1}\left(T\right)-1$$

where *θ_c_* is the apparent contact angle in the Cassie state, *θ*_0_ is the intrinsic contact angle, and *f*_1_ is the solid-liquid contact area fraction of the substrate.

**Figure 7:**
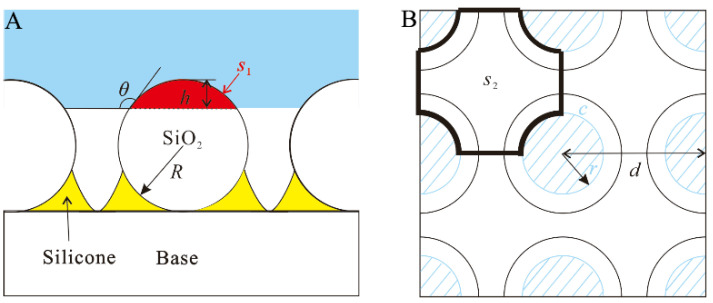
Schematic diagram of microstructure-supported droplets. (**A**) Front view; (**B**) top view.

Figure 7 shows a schematic diagram of the microstructure-supported droplet. In the three-dimensional topography, it can be found that the radius *R* of the microspheres formed by the aggregation of the hydrophobic nano-SiO_2_ particles sprayed on the surface is about 2.5 μm. From the advancing contact angle data, it can be obtained that the depth *h* when the droplet infiltrates the microsphere is 0.43 μm and the radius *r* of the wetting ball section is 1.97 μm. At this time, the area of the microsphere crown *S*_1_ is 6.77 μm^2^, and the three-phase contact wire length *c* is 12.38 μm.

According to the critical three-phase contact line density, the critical point from the Wenzel state to the Cassie state is obtained, and the center distance *d* between the microspheres is 5.13 μm. At this time, the gas-liquid contact area *S*_2_ is 14.08 μm^2^, and the theoretical maximum contact angle size obtained by substituting the data into Equation (3) is 150°.

When a layer of microspheres completely covers the surface, *d* is 5 μm. At this time, the gas-liquid contact area fraction of the Cassie state is the smallest, forming the minimum contact angle of the superhydrophobic surface Cassie state. Substituting the data into Equation (3) calculates that the droplet contact angle is 149° when it is completely flattened.

When the hydrophobic nano-SiO_2_ particles have completely tiled the surface and reach saturation, the excess hydrophobic nano-SiO_2_ particles will be stacked on the three-dimensional level, and the solid-liquid contact area will be smaller in the process of random stacking, which leads to a larger contact angle of the superhydrophobic surface. Due to the characteristics of the spraying process, the same surface will be sprayed multiple times. The ideal tiled Cassie state will not appear. During the spraying process, it should be in the form of a stacked Cassie state.

## 4. Conclusions

In this paper, the influence of the concentration of hydrophobic nano-SiO_2_ in the coating on the wetting performance of the coating was studied, and the change of the wetting state of the substrate surface with the increase of the concentration of the hydrophobic nano-SiO_2_ was analyzed through a theoretical model. When the concentration of hydrophobic nano-SiO_2_ in the coating is higher than 0.05 g/mL, the contact angle of the coating surface sprayed on the polished substrate is greater than 150°, but if the concentration is too high, cracks will appear on the surface of the coating and reduce the mechanical properties, so the suitable hydrophobic nano-SiO_2_ concentration is 0.05 g/mL. The prepared superhydrophobic surface has low adhesion to droplets, mainly because the microstructure can effectively encapsulate air, reducing the solid-liquid contact area, and thereby reducing the adhesion between the solid-liquid interface. Theoretical analysis shows that there are three wetting states in the spraying process: Wenzel state, tiled Cassie state, and stacked Cassie state. When the substrate surface enters the Cassie state, the lower limit of the contact angle is 149°. This work provided a promising conception for advancing the practical application of superhydrophobic surfaces.

## Data Availability

Not applicable.
